# Als3 Is a Candida albicans Invasin That Binds to Cadherins and Induces Endocytosis by Host Cells

**DOI:** 10.1371/journal.pbio.0050064

**Published:** 2007-02-20

**Authors:** Quynh T Phan, Carter L Myers, Yue Fu, Donald C Sheppard, Michael R Yeaman, William H Welch, Ashraf S Ibrahim, John E Edwards, Scott G Filler

**Affiliations:** 1 Department of Medicine, Los Angeles Biomedical Research Institute, Harbor-UCLA Medical Center, Torrance, California, United States of America; 2 David Geffen School of Medicine, University of California Los Angeles, Los Angeles, California, United States of America; 3 Department of Microbiology and Immunology, McGill University, Montreal, Quebec, Canada; 4 Department of Biochemistry, University of Nevada Reno, Reno, Nevada, United States of America; Duke University Medical Center, United States of America

## Abstract

Candida albicans is the most common cause of hematogenously disseminated and oropharyngeal candidiasis. Both of these diseases are characterized by fungal invasion of host cells. Previously, we have found that C. albicans hyphae invade endothelial cells and oral epithelial cells in vitro by inducing their own endocytosis. Therefore, we set out to identify the fungal surface protein and host cell receptors that mediate this process. We found that the C. albicans Als3 is required for the organism to be endocytosed by human umbilical vein endothelial cells and two different human oral epithelial lines. Affinity purification experiments with wild-type and an *als3*Δ/*als3*Δ mutant strain of C. albicans demonstrated that Als3 was required for C. albicans to bind to multiple host cell surface proteins, including N-cadherin on endothelial cells and E-cadherin on oral epithelial cells. Furthermore, latex beads coated with the recombinant N-terminal portion of Als3 were endocytosed by Chinese hamster ovary cells expressing human N-cadherin or E-cadherin, whereas control beads coated with bovine serum albumin were not. Molecular modeling of the interactions of the N-terminal region of Als3 with the ectodomains of N-cadherin and E-cadherin indicated that the binding parameters of Als3 to either cadherin are similar to those of cadherin–cadherin binding. Therefore, Als3 is a fungal invasin that mimics host cell cadherins and induces endocytosis by binding to N-cadherin on endothelial cells and E-cadherin on oral epithelial cells. These results uncover the first known fungal invasin and provide evidence that C. albicans Als3 is a molecular mimic of human cadherins.

## Author Summary

The fungus Candida albicans is usually a harmless colonizer of human mucosal surfaces. In the mouth, it can cause oropharyngeal candidiasis, also called thrush. In hospitalized and immunocompromised patients, C. albicans can enter the blood stream and be carried throughout the body to cause a disseminated infection, which is associated with a mortality rate of up to 40%. The organism invades the epithelial cell lining of the mouth during oropharyngeal candidiasis and invades the endothelial cell lining of the blood vessels during disseminated candidiasis. We discovered that Als3, a protein expressed on the surface of *C. albicans,* is required for this invasion process. Cadherins on the surface of human cells normally bind other cadherins for adhesion and signaling; however, we found that Als3 also binds to cadherins on endothelial cells and oral epithelial cells, and this binding induces these host cells to take up the fungus. The structure of Als3 is predicted to be quite similar to that of the two cadherins studied, and the parameters of the binding of Als3 to either cadherin are similar to those of cadherin–cadherin binding. These results suggest that Als3 is a functional and structural mimic of human cadherins, and provide new insights into how C. albicans invades host cells.

## Abstract 3

Als3 aids the invasion of the fungal pathogen*Candida albicans* into human host cells by mimicking human cadherins to induce endocytosis.

## Introduction

The fungus Candida albicans causes both hematogenously disseminated and oropharyngeal disease. During the initiation of hematogenously disseminated candidiasis, blood-borne organisms invade the endothelial cell lining of the vasculature to infect deeper tissues. Fungal invasion of the superficial oral epithelial cells is also characteristic of oropharyngeal candidiasis [1–3]. The invasion of either endothelial or oral epithelial cells by C. albicans is necessary for the organism to damage these cell types in vitro [4,5]. Host cell invasion and damage are likely critical virulence attributes of C. albicans because mutants with defects in these processes in vitro are highly likely to have attenuated virulence in experimental animal models of hematogenously disseminated or oropharyngeal candidiasis [5–8].

Because of the importance of host cell invasion in the pathogenesis of candidiasis, we have been investigating the mechanism by which this process occurs. Previously, we have found that C. albicans invades both endothelial cells and oral epithelial cells in vitro by inducing its own endocytosis [4,5,7]. Endothelial cell endocytosis of C. albicans is induced when the organism binds to N-cadherin and other endothelial cell surface proteins. This binding induces microfilament rearrangement, which results in the formation of pseudopods that engulf the organism and draw it into the cell [9,10]. Endothelial cell endocytosis of C. albicans is dependent on extracellular calcium and is governed at least in part by the tyrosine phosphorylation of endothelial cell proteins [9,11].

C. albicans is a dimorphic fungus that can grow as either ovoid-shaped blastospores or as filamentous hyphae. The ability to reversibly change between blastospores and hyphae is a key virulence factor for this fungus, and mutants that are unable to form hyphae have greatly attenuated virulence [12,13]. We have found previously that C. albicans mutants with defects in the signal transduction pathways that govern hyphal formation have a significantly reduced capacity to invade both endothelial and oral epithelial cells in vitro [5–7]. Furthermore, studies with killed organisms indicate that induction of endocytosis is passive on the part of the organism because killed hyphae are endocytosed as avidly as are live hyphae [5,7].

An important, yet-unanswered question from these studies has been the identity of the C. albicans surface structure(s) that induces host cell endocytosis. Numerous other medically important fungi such as *Aspergillus fumigatus, Cryptococcus neoformans,* and Rhizopus oryzae invade normally nonphagocytic host cells [14–18]. Remarkably, no fungal surface protein that induces fungal uptake by these host cells has been identified. Discovering such an invasin-like protein is important not only for its biological significance, but also because it could serve as a therapeutic or vaccine target.

In bacterial pathogens, proteins that mediate host cell invasion frequently also function as adhesins [19]. In *C. albicans,* a major group of adhesins consists of the proteins encoded by the *ALS* gene family. This gene family encodes eight GPI-linked cell surface proteins that mediate binding to diverse host substrates [20–25]. Each Als protein has three domains. The N-terminal domain contains the substrate-binding region [23,26]. The central portion of the protein consists of a variable number of 36–amino acid tandem repeats. The C-terminal domain is rich in serine and threonine, and contains a GPI anchorage sequence that is predicted to be cleaved as the protein is exported to the cell surface [20].

Computer-assisted modeling of the N-termini of Als1, Als3, and Als5 predicts the presence of antiparallel β sheets that define the immunoglobulin superfamily [23]. This prediction has been substantiated by circular dichroism and Fourier transform infrared spectroscopic analysis of the N-terminus of Als1 and Als5 [23,27]. Interestingly, the predicted 3-D structures of the N-termini of many Als proteins are remarkably similar to the Yersinia pseudotuberculosis invasin, a protein that induces the endocytosis of Y. pseudotuberculosis by mammalian epithelial cells [28,29]. This relationship led us to investigate whether any of the Als proteins could induce host cell endocytosis. We found that Saccharomyces cerevisiae expressing Als3 were avidly endocytosed by endothelial cells. Similar experiments suggested that Als1 and Als5 could also induce endocytosis, although with considerably less efficiency than Als3 [23].

In the current study, we investigated the role of Als1 and Als3 in inducing the endocytosis of C. albicans by both endothelial and oral epithelial cells. We discovered that Als3, and to a lesser extent Als1, induce endocytosis of C. albicans by binding to N-cadherin on endothelial cells and E-cadherin on oral epithelial cells. Furthermore, computer-assisted molecular modeling of the binding of Als3 with N- and E-cadherin suggests that Als3 is a structural and functional mimic of these cadherins.

## Results

### Als3 Is Required for C. albicans to Be Endocytosed by Endothelial Cells and Oral Epithelial Cells

**Figure 1:**
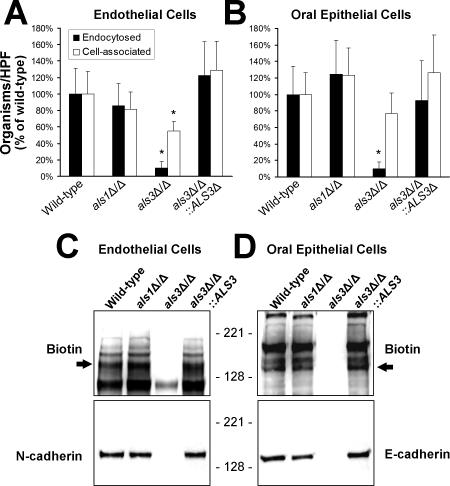
An *als3*Δ/*als3*Δ Mutant of C. albicans Is Endocytosed Poorly by Endothelial and Oral Epithelial Cells, and Does Not Bind to N-Cadherin or E-Cadherin (A and B) Hyphae of the indicated strains of C. albicans were incubated for 45 min with human umbilical vein endothelial cells (A) or the FaDu oral epithelial cell line (B), after which the number of endocytosed and cell-associated (endocytosed plus adherent) organisms was determined by a differential fluorescence assay. The data are expressed as a percentage of the results with wild-type organisms, and are the mean ± SD of three experiments, each performed in triplicate. *, *p* < 0.001 compared to the wild-type strain and the *als3*Δ/*als3*Δ::*ALS3*-complemented strain. (C and D) Binding of surface proteins from endothelial cells (C) and FaDu oral epithelial cells (D) to hyphae of the indicated strains of C. albicans. Biotinylated endothelial or epithelial cell membrane proteins that bound to C. albicans were eluted from the hyphae with urea and separated by SDS-PAGE. (C) The same immunoblot of endothelial cell surface proteins was developed with an anti-biotin monoclonal antibody (top) and an anti-N-cadherin monoclonal antibody (bottom). (D) An immunoblot of oral epithelial cell surface proteins was developed with an anti-biotin monoclonal antibody (top) and an anti-E-cadherin monoclonal antibody (bottom). Arrows in (C) and (D) indicate the bands that correspond to N-cadherin and E-cadherin, respectively.

The interactions of *als1*Δ/*als1*Δ and *als3*Δ/*als3*Δ mutant strains of C. albicans with human umbilical vein endothelial cells and the FaDu oral epithelial cell line were investigated. Using our standard differential fluorescence assay, we determined the capacity of each strain of C. albicans to adhere to these host cells and induce its own endocytosis. Hyphae of the *als1*Δ/*als1*Δ null mutant interacted with both host cell types similarly to the wild-type strain (Figure 1A and 1B). In contrast, there was a 90% reduction in the number of hyphae of the *als3*Δ/*als3*Δ mutant that were endocytosed by either endothelial or oral epithelial cells compared to the wild-type control strain. Also, there was a significant reduction in the number of hyphae of the *als3*Δ/*als3*Δ mutant that were cell-associated with endothelial cells, but not oral epithelial cells. These results suggest that Als3 contributes to adherence to endothelial cells. We verified that the reduced endocytosis and adherence of the *als3*Δ/*als3*Δ mutant was due to the absence of *ALS3,* because complementing this mutant with a wild-type copy of *ALS3* restored endocytosis and adherence to wild-type levels.

### Als3 Is Necessary for C. albicans to Bind to N-Cadherin on Endothelial Cells and E-Cadherin on Oral Epithelial Cells

Endocytosis of C. albicans by endothelial cells is induced when the organism binds to N-cadherin on the endothelial cell surface [9]. We used an affinity purification approach to determine whether Als1 or Als3 was required for C. albicans hyphae to bind to N-cadherin in endothelial cell membrane extracts. As predicted by the results of the endocytosis assay, the *als1*Δ/*als1*Δ mutant bound to the same endothelial cell surface proteins, including N-cadherin, as did the wild-type strain (Figure 1C). In contrast, the *als3*Δ/*als3*Δ null mutant did not bind to N-cadherin, and it bound very weakly to the other endothelial cell surface proteins. This defect in binding to N-cadherin was reversed when the *als3*Δ/*als3*Δ null mutant was complemented with a wild-type copy of *ALS3*. Therefore, Als3 is required for C. albicans to bind to N-cadherin and other proteins on the endothelial cell surface.

The surface proteins on oral epithelial cells that are bound by C. albicans hyphae have not been identified previously. We found that the wild-type strain of C. albicans bound to at least four different proteins on the surface of FaDu oral epithelial cells (Figure 1D). Many of these proteins appeared to be different from the endothelial cell proteins that were bound by wild-type *C. albicans.* FaDu oral epithelial cells express very low levels of N-cadherin (unpublished data), but high levels of E-cadherin. Therefore, we investigated whether E-cadherin was one of the epithelial cell proteins that was bound by *C. albicans.* Using an anti-E-cadherin monoclonal antibody, we detected a significant amount of this protein in immunoblots of epithelial cell membrane proteins that were bound by hyphae of wild-type C. albicans (Figure 1D). Furthermore, although the *als1*Δ/*als1*Δ mutant still bound to E-cadherin, the *als3*Δ/*als3*Δ null mutant did not.

To verify that these results were not specific to the FaDu oral epithelial cell line, we repeated these experiments using the OKF6/TERT-2 cell line. This cell line was developed by the forced expression of the human telomerase catalytic subunit in oral mucosal keratinocytes obtained from a healthy adult [30]. The *als3*Δ/*als3*Δ null mutant was endocytosed poorly by OKF6/TERT-2 cells and bound poorly to the surface proteins, including E-cadherin, of this cell line (Figure S1). In contrast, wild-type C. albicans bound to similar surface proteins of both OKF6/TERT-2 and FaDu epithelial cells. Collectively, these results indicate that C. albicans hyphae bind to epithelial cell E-cadherin in an Als3-dependent manner.

When C. albicans interacts with the oral epithelium in vivo, the organisms are coated with saliva, which has been reported to either inhibit or enhance the adherence of C. albicans to oral epithelial cells [31,32]. Also, it is possible that salivary proteins could potentially act as bridging molecules between the hyphae and oral epithelial cells and thereby facilitate endocytosis of C. albicans. To investigate these possibilities, we incubated C. albicans hyphae in the presence and absence of 20% saliva and then measured their interactions with both intact FaDu oral epithelial cells and oral epithelial cell surface proteins. Killed organisms were used in these experiments to obviate potential effects of saliva on the growth of the hyphae. We have previously shown that killed hyphae adhere to and are endocytosed by oral epithelial cells similarly to live hyphae [5]. Incubating hyphae in saliva prior to adding them to the epithelial cells had no effect on the number of endocytosed or cell-associated organisms (Figure S2A). As predicted by the endocytosis assay, saliva also had no detectable effect on the binding of epithelial cell surface proteins, including E-cadherin, to C. albicans hyphae (Figure S2B and S2C). Therefore, salivary components do not act as bridging molecules between the organisms and epithelial cells.

**Figure 2:**
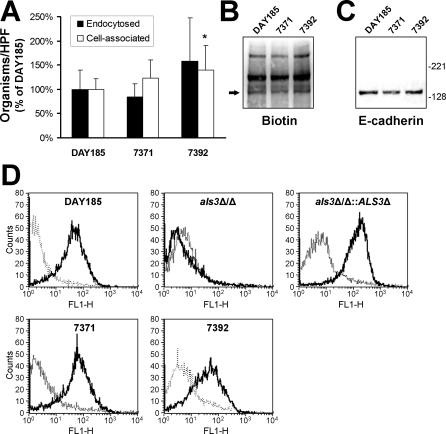
Endocytosis and E-Cadherin Binding of Different Strains of C. albicans (A) Number of hyphae of the indicated clinical isolates of C. albicans that were endocytosed by and cell-associated with the FaDu oral epithelial cell line after a 45-min incubation. The data are expressed as a percentage of the results with strain DAY185, and are the mean ± SD of three experiments, each performed in triplicate. *, *p* < 0.05 compared to strain DAY185. (B and C) Immunoblot of biotinylated FaDu cell surface proteins eluted from the indicated strains of *C. albicans.* The same blot was probed with antibodies against biotin (B) or E-cadherin (C). (D) Flow cytometric detection of Als3 expression on the surface of C. albicans hyphae. Hyphae of the indicated strains were stained using indirect immunofluorescence with either a polyclonal rabbit anti-Als3 antiserum (solid lines) or purified rabbit IgG (broken lines) and then analyzed using flow cytometry.

Next, we confirmed that oral isolates of C. albicans other than those derived from the oral strain SC5314 also bound to E-cadherin and were endocytosed by FaDu oral epithelial cells. Two additional strains that had been obtained from patients with AIDS who had oropharyngeal candidiasis were tested in our assays. Hyphae of these strains were endocytosed similarly by the oral epithelial cells, although strain 7392 had slightly higher adherence to these cells (Figure 2A). Also, all strains bound to the same oral epithelial cell surface proteins, including E-cadherin (Figure 2B and 2C). To verify that that these strains expressed Als3 on their surface, we used flow cytometry with an Als3-specific polyclonal antiserum. As expected, all wild-type strains had detectable Als3 on their surface, whereas the *als3*Δ/*als3*Δ null mutant did not (Figure 2D).

### Colocalization of N-Cadherin and E-Cadherin with Endocytosed C. albicans Hyphae Requires Als3

Next, we verified that binding of C. albicans hyphae to N-cadherin and E-cadherin on the surface of intact host cells required Als3 and was associated with induction of endocytosis. Monolayers of endothelial cells or FaDu oral epithelial cells were infected with the various strains of *C. albicans,* after which N-cadherin and E-cadherin were detected by indirect immunofluorescence. To determine if the organisms were in the process of being endocytosed, the host cells were also stained for f-actin, which accumulates around such organisms [4,5].

**Figure 3:**
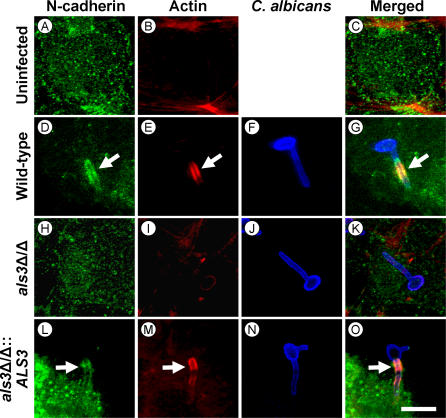
Endothelial Cell N-Cadherin Colocalizes with Wild-Type *C. albicans,* but Not an *als3*Δ/*als3*Δ Mutant Strain Confocal micrographs of uninfected endothelial cells (A–C), or endothelial cells infected with the wild-type strain (D–G), the *als3*Δ/*als3*Δ null mutant (H–K), or the *als3*Δ/*als3*Δ::*ALS3*-complemented strain (L–O). The cells were stained for N-cadherin (A), (D), (H), and (L), actin microfilaments (B), (E), (I), and (M), and C. albicans (F), (J), and (N). The merged images are shown in (C), (G), (K), and (O). Arrows indicate the accumulation of N-cadherin and microfilaments around the organisms. Bar in (O) indicates 10 μm.

**Figure 4:**
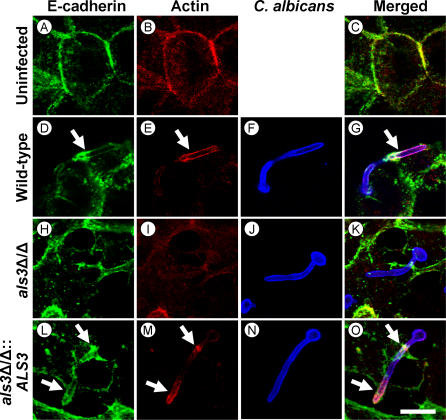
E-Cadherin from Oral Epithelial Cells Colocalizes with Wild-Type *C. albicans,* but Not an *als3*Δ/*als3*Δ Mutant Strain Confocal micrographs of uninfected FaDu oral epithelial cells (A–C) or epithelial cells infected with the wild-type strain (D–G), the *als3*Δ/*als3*Δ null mutant (H–K), or the *als3*Δ/*als3*Δ::*ALS3*-complemented strain (L–O). The cells were stained for E-cadherin (A), (D), (H), and (L), microfilaments (B), (E), (I), and (M), and C. albicans (F), (J), and (N). The merged images are shown in (C), (G), (K), and (O). Arrows indicate the accumulation of E-cadherin and microfilaments around the organisms. Bar in (O) indicates 10 μm.

We observed that endothelial cell N-cadherin colocalized with hyphae of the wild-type strain and the *als1*Δ/*als1*Δ null mutant that were being endocytosed (Figure 3). In contrast, N-cadherin did not colocalize with hyphae of the *als3*Δ/*als3*Δ null mutant, and almost none of these hyphae were endocytosed. As expected, N-cadherin accumulated around hyphae of the *als3*Δ/*als3*Δ::*ALS3* complemented strain similarly to the wild-type control strain. A comparable pattern was observed with E-cadherin and oral epithelial cells (Figure 4). E-cadherin colocalized with the endocytosed hyphae of all strains except for the *als3*Δ/*als3*Δ null mutant. Also, very few hyphae of the *als3*Δ/*als3*Δ mutant were endocytosed. The anti-N-cadherin and anti-E-cadherin monoclonal antibodies did not bind to C. albicans hyphae in the absence of endothelial or epithelial cells (Figure S3), indicating that these antibodies did not recognize any cross-reacting C. albicans antigens. Collectively, these findings support a model in which C. albicans Als3 binds to endothelial cell N-cadherin and oral epithelial cell E-cadherin, thereby stimulating actin-mediated endocytosis of the organism.

### Als3 Is Required for C. albicans to Damage Endothelial Cells and Oral Epithelial Cells

**Figure 5:**
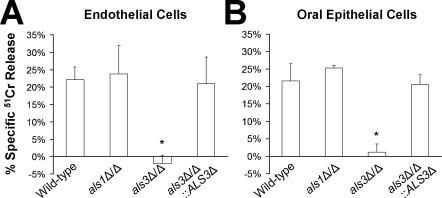
An *als3*Δ/*als3*Δ Mutant of C. albicans Has Reduced Capacity to Damage Endothelial Cells and Oral Epithelial Cells Endothelial cells or FaDu oral epithelial cells were incubated with the indicated strains for 180 min, after which the extent of host cell damage was determined by a ^51^Cr release assay. The data are the mean ± SD of three experiments, each performed in triplicate. *, *p* < 0.0001 compared to the wild-type strain and the *als3*Δ/*als3*Δ::*ALS3*-complemented strain.

Infection of endothelial cells or oral epithelial cells with wild-type C. albicans hyphae in vitro causes significant damage to these cells, and endocytosis of the organisms is required to induce host cell damage [4,5,10]. Therefore, we investigated the extent of damage to endothelial cells and oral epithelial cells caused by the *als1*Δ/*als1*Δ and *als3*Δ/*als3*Δ null mutants. Consistent with the results of the endocytosis assay, the *als1*Δ/*als1*Δ null mutant caused the same amount of damage to both types of host cell as the wild-type strain (Figure 5). In contrast, the *als3*Δ/*als3*Δ null mutant caused essentially no damage to either cell type. Complementing the *als3*Δ/*als3*Δ null mutant with a wild-type copy of *ALS3* restored its capacity to damage these host cells. These data indicate that the inability of the *als3*Δ/*als3*Δ null mutant to invade endothelial cells and oral epithelial cells is associated with a significantly reduced capacity to damage these cells.

### Als1 and Als3 by Themselves Are Sufficient to Induce Endocytosis

**Figure 6:**
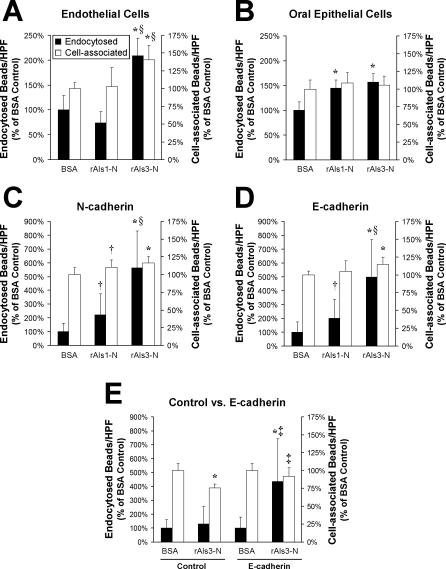
Adherence and Endocytosis of Latex Beads Coated with the N-Terminal Portion of Als1 or Als3 (A–D) Latex beads were coated with BSA, rAls1-N, or rAls3-N. They were incubated for 45 min with endothelial cells (A), FaDu oral epithelial cells (B), CHO cells expressing human N-cadherin (C), or CHO cells expressing human E-cadherin (D), after which the number of endocytosed and cell-associated beads was determined. (E) The interactions of BSA- and rAls3-N–coated beads with control CHO cells expressing no human cadherins (Control) were compared with those of CHO cells expressing human E-cadherin (E-cadherin) by the same method as in (A–D). Data are expressed as a percentage of the results with beads coated with BSA and are the mean ± SD of three or four experiments, each performed in triplicate. *, *p* ≤ 0.005 compared to beads coated with BSA; †*p* < 0.05 compared to beads coated with BSA; §*p* < 0.005 compared to beads coated with rAls1-N; and ‡*p* ≤ 0.01 compared to control CHO cells.

To determine if either Als1 or Als3 alone was able to induce endocytosis, we coated latex beads with the purified, recombinant N-terminal portion of Als1 (rAls1-N) or Als3 (rAls3-N), which consisted of amino acids 17 to 432 of either protein. Control beads were coated with biotinylated bovine serum albumin (BSA). We then assessed the number of beads that were endocytosed by and associated with endothelial cells and FaDu oral epithelial cells. Although rAls1-N–coated beads were endocytosed similarly to the BSA-coated beads by endothelial cells, significantly more of these beads were endocytosed by FaDu epithelial cells (Figure 6A and 6B). The number of rAls1-N–coated beads that were associated with both cell types was similar to that of the BSA-coated beads, indicating that the N-terminal portion of Als1 by itself is insufficient to mediate adherence to either cell type. Beads coated with rAls3-N were endocytosed avidly by both endothelial and oral epithelial cells. Also, a greater number of beads coated with Als3 were associated with the endothelial cells compared with the control beads.

Next, we examined the interactions of the beads with Chinese hamster ovary (CHO) cells expressing N-cadherin or E-cadherin to investigate whether binding of Als1 or Als3 to these cadherins alone was sufficient to induce endocytosis. The CHO cell assay was more sensitive than the assays using endothelial cells or oral epithelial cells because the background adherence and endocytosis of the BSA-coated beads was lower when CHO cells were used (unpublished data). Beads coated with rAls1-N were endocytosed more efficiently than the control beads by CHO cells expressing either N-cadherin or E-cadherin (Figure 6C and 6D). Also, slightly more rAls1-N–coated beads were associated with the CHO cells expressing N-cadherin, but not the ones expressing E-cadherin. However, the beads coated with rAls3-N were endocytosed significantly more efficiently by the CHO cells expressing N- or E-cadherin than were the beads coated with either BSA or rAls1-N. In addition, a slightly greater number of rAls3-N–coated beads were associated with cells expressing either N-cadherin or E-cadherin than were the control beads. The enhanced endocytosis mediated by rAls3-N required the presence of either N- or E-cadherin on the surface of the CHO cells because beads coated with rAls3-N were endocytosed similarly to beads coated with BSA by control CHO cells that did not express either N- or E-cadherin (Figure 6E). These results demonstrate that Als3, and to a lesser extent Als1, bind directly to N-cadherin and E-cadherin, and that this binding is sufficient to induce endocytosis. These data also indicate that the cadherin-binding domains of Als1 and Als3 are located in the N-termini of these proteins.

### The Predicted N-Terminal Structures of Als1 and Als3 Are Different

**Figure 7:**
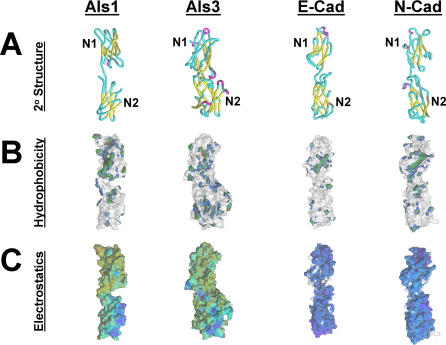
Comparison of the First Two N-Terminal Domains of Als1, Als3, E-Cadherin, and N-Cadherin The most distal (N1) and adjacent (N2) N-terminal domains of Als1, Als3, E-cadherin, and N-cadherin are shown. The predicted structures of the N-terminal regions of Als1 and Als3 were extracted from their larger models, and structures for E-cadherin and N-cadherin were obtained from the Protein Data Bank [62]. (A) Comparative secondary structures. Yellow color indicates β-sheet propensity; red, α-helical propensity; aqua, extended or turn structures. Note that the Als1 model encompasses 187 amino acids (residues 3–190) in an equivalent 3-D space as that of Als3, which includes 279 amino acids (residues 20–299). Such a comparison illustrates the overall similarities in conformation among these polypeptide domains, with a more compact configuration of Als3. (B) Comparative hydrophobic surfaces. Surface hydrophobicity is illustrated in color upon the solvent-accessible surface areas of respective molecules. For clarity, only the hydrophobic surface is colored; the ghost structures (gray) represent the polar surface. In hydrophobic areas, brown indicates the most hydrophobic regions and blue indicates the least hydrophobic regions. (C) Comparative electrostatic surfaces. Negatively charged electrostatic surface is illustrated in color upon the solvent accessible surface areas of respective molecules. For clarity, only the negatively charged surface is colored; the ghost structures (gray) represent the small area on each molecule bearing a positive charge. In negatively charged areas blue indicates the strongest negative surface potential and yellow indicates the weakest negative charge potential.

**Figure 8:**
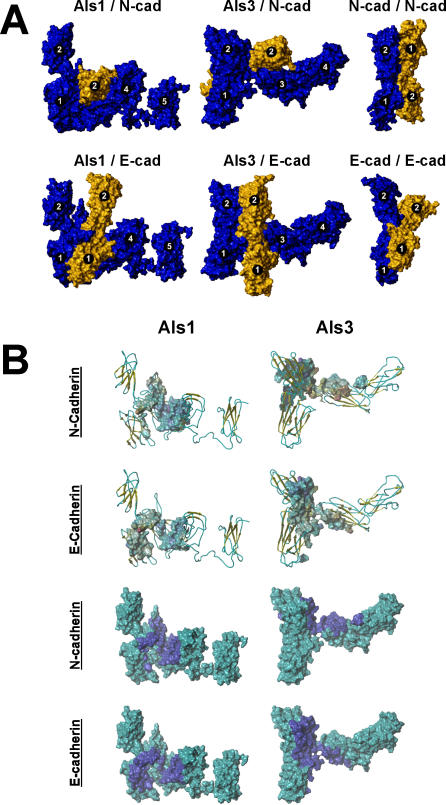
Comparative Models of Als–Cadherin Interactions The interactions of the N-terminal regions of Als1 and Als3 with the N-terminal regions of N-cadherin and E-cadherin, as well as N-cadherin and E-cadherin self-association were modeled as outlined in Materials and Methods. The N-terminal domains of Als1 and Als3 are shown in their energy-minimized conformations; docking results using alternate conformations yielded equivalent results (unpublished data). (A) The solvent-accessible surface areas of the proteins are shown, and the β barrel domains are numbered sequentially from the N-terminus. In models of the docking of an Als protein to a cadherin (left and middle columns), the Als protein is blue and the cadherin is gold. In models of cadherin self-association, one cadherin molecule is blue and the other cadherin is gold. (B) Interactions of Als1 or Als3 with E-cadherin or N-cadherin are presented to illustrate their predicted docking characteristics. In the top two rows, the indicated cadherin electrostatic contact surfaces are superimposed upon the backbone structures of Als1 or Als3. Coloration schema of the Als secondary structure: gold, β sheet; blue, extended/turn. Coloration schema of the cadherin surface: blue, most negative; red, most positive. In the bottom two rows, the contact footprints of the indicated cadherins (dark blue) are superimposed upon the solvent-accessible surface areas of Als1 or Als3 (aqua) to indicate the predicted docking positions of the protein pairs. In all cases, note that the ectodomain N2 of Als3 contributes to the interactions with both cadherins, while the corresponding ectodomain N2 of Als1 does not participate in interactions with either cadherin.

Although the N-terminal regions of Als1 and Als3 are 84% identical at the amino acid level, they interacted differently with N- and E-cadherin. Therefore, we used computer-assisted modeling to identify differences in the secondary structure of the N-terminal regions of Als1 and Als3 that might account for their functional differences. Our initial models of the N-terminal regions of these proteins indicated that they share general structural features, including a modular β sheet configuration interposed by extended regions, and an overall negative surface potential [23]. In the current study, we generated higher resolution models of the N-terminal regions of Als1 and Als3. These models predicted that the N-termini of both proteins contain recurrent β barrel (“beads-on-a-string”) domains (Figure 7A). Interestingly, the N-terminal regions of Als1 and Als3 bear a striking resemblance to the N-terminal ectodomains of N- and E-cadherin. The cadherin ectodomains have five β barrel domains, the first two of which are shown in Figure 7A. Similarly, the N-terminal regions of Als1 and Als3 contain at least four β barrel domains (the first two of these domains are shown in Figure 7A, and all four domains are shown in Figure 8). Notably, the first such domain (N1 domain) of each of the four proteins contains their most hydrophobic sections (Figure 7B). However, Als1 and Als3 differ from the cadherins in that the fungal proteins bear less negative charge on their N1 than their N2 domains, whereas the cadherins bear the most negative charge on their N1 domains (Figure 7C).

The present computational models of the Als1 and Als3 N-terminal regions also indicated potentially significant differences between these two proteins. The most conspicuous differences are the predicted size and shape of these regions. Both Monte Carlo and molecular dynamics simulations indicate that the N-terminal domains of Als1 favor an extended conformation, while those of Als3 tend toward a more compact, β propeller–like arrangement (Figure 8). Also, the spatial distribution of surface hydrophobicity and electrostatic potential within the N1 and N2 domains differ between Als1 and Als3 (Figure 7B and 7C).

### Als3 Is Predicted to Bind to Cadherins Differently than Als1

Next, we modeled potential sites of interaction between the N-terminal regions of Als1, Als3, N-cadherin, and E-cadherin. The N-terminal domains of Als1 and Als3 were modeled in common, semi-extended conformations (Figure 8). This consensus conformation allowed comparable access of any potential Als-docking domains to the cadherin ligands. Significant differences between Als1 and Als3 docking interactions with N- and E-cadherin were found. The binding of Als1 to N-cadherin was predicted to have greater contact area and electrostatic energy compared to the binding of Als3 to N-cadherin (Table 1). The hydrophobicity and hex energy of the binding of either Als1 or Als3 to N-cadherin were similar. However, the overall favorability of binding to N-cadherin was much better for Als3 than for Als1. Interestingly, the predicted parameters of Als3 binding to N-cadherin were very similar to those of one molecule of N-cadherin binding to another (N-cadherin self-association). The docking studies also predicted that different regions of Als1 and Als3 bind to N-cadherin. The N1 and N3 domains of Als1 bind to domains N1 and N2 of N-cadherin, whereas the N2 and N3 domains of Als3 bind to domains N1 and N2 (Table 1 and Figure 8A).

**Table 1:**
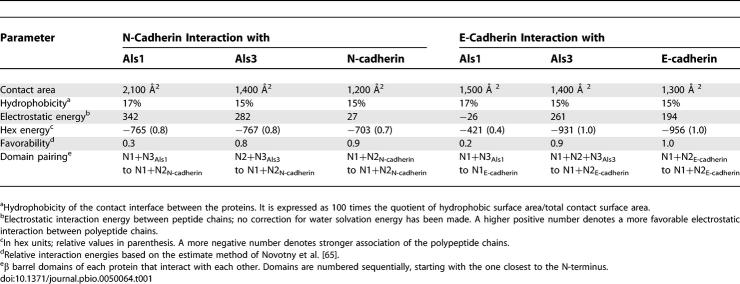
Relative Physicochemical Contributions to Binding of N-Terminal Regions of Als1 and Als3 to N-Cadherin and E-Cadherin

The N-terminal regions of Als1 and Als3 are also predicted to interact differently with E-cadherin. The energetics of Als1 binding to E-cadherin are considerably weaker than either Als3 binding to E-cadherin or E-cadherin binding to itself (Table 1). Also, Als1 N1 and N3 domains likely interact only with the N1 domain of E-cadherin, and they do not appear to have energetically favorable interactions with the N2 domain of this cadherin (Table 1 and Figure 8). Als3 is predicted to interact with E-cadherin differently than Als1. Moreover, the Als3-E-cadherin interaction is somewhat different from the Als3–N-cadherin interaction. Domains N1, N2, and N3 of Als3 appear to form a cleft that binds E-cadherin domains N1 and N2 (Figure 8). More important, the geometry of this interaction assumes a parallel orientation, such as that which occurs when E-cadherin binds to itself (Figure 8A).

## Discussion

Previous studies have focused on the adherence properties of the Als proteins [23–25,33–38]. The different Als proteins mediate adherence to broad variety of host substrates, and this adherence is likely critical for C. albicans to infect host surfaces. In this study, we report that an additional function of Als3 is that it can act as an invasin protein and induce endocytosis by normally nonphagocytic host cells. Furthermore, we show that this endocytosis occurs as a result of the N-terminal region of Als3 binding to either N-cadherin or E-cadherin. These conclusions are supported by the following results. First, an *als3*Δ/*als3*Δ null mutant was endocytosed poorly by endothelial cells and two different oral epithelial cell lines. Second, latex beads coated with rAls3-N were efficiently and specifically endocytosed by both endothelial cells and oral epithelial cells. Third, the rAls3-N–coated beads were avidly endocytosed by CHO cells expressing either N-cadherin or E-cadherin, but not by CHO cells that did not express these cadherins. These current results are also consistent with our previous data that S. cerevisiae expressing Als3 are endocytosed by endothelial cells [23].

In our prior studies, we have determined that C. albicans must be endocytosed by either endothelial cells or oral epithelial cells to cause damage to these cells [4,5,39]. We found that the *als3*Δ/*als3*Δ null mutant caused virtually no damage to either endothelial cells or FaDu oral epithelial cells. Similarly, Zhao et al. [24] reported that an *als3*Δ/*als3*Δ null mutant had markedly reduced capacity to damage reconstituted human epithelium. Our results suggest that the reduced capacity of the *als3*Δ/*als3*Δ null mutant to damage endothelial cells and oral epithelial cells in vitro is due to its defect in invading these cells.

The invasion and damage defects of the *als3*Δ/*als3*Δ null mutant suggest that Als3 is important for virulence during hematogenously disseminated and oropharyngeal candidiasis. Investigations to determine the role of Als3 in C. albicans virulence are currently ongoing. However, we have found that vaccination of mice with rAls3 provides significant protection from lethal hematogenously disseminated candidiasis and ameliorates the course of oropharyngeal and vaginal candidiasis [40]. Thus, Als3 is a useful therapeutic target.

In our assays, the *als3*Δ/*als3*Δ null mutant had significantly reduced adherence to endothelial cells, but normal adherence to oral epithelial cells. It has been reported by others that an *als3*Δ/*als3*Δ null mutant had reduced adherence to endothelial cells, buccal epithelial cells, and FaDu epithelial cells [24,35]. Also, we found that S. cerevisiae expressing Als3 bound to both endothelial cells and FaDu oral epithelial cells [23]. The discrepancy between the previous and current results is likely due to differences in the methodology of the assays. Specifically, the longer incubation time and the use of a 24-well tissue culture plate (rather than a 6-well plate) in the current endocytosis assay make it less sensitive to differences in adherence among strains.

We also found that the *als1*Δ/*als1*Δ mutant was endocytosed normally by oral epithelial cells, whereas latex beads coated with rAls1-N induced some endocytosis by these cells. The probable explanation for these apparently conflicting results is that the *als1*Δ/*als1*Δ mutant still expressed Als3 on its surface. Thus, the presence of Als3 masked the effects of the absence of Als1 when the endocytosis assay was performed using whole organisms. Studies performed with latex beads coated with rAls1-N indicated that Als1 is capable of inducing epithelial cell endocytosis by itself. Collectively, these results demonstrate the utility of using both mutant analysis and recombinant proteins to assess the contribution of an individual Als protein to host cell interactions.

Previously, we determined that the N-terminal region of Als proteins contains the substrate-binding site [23,26]. This conclusion is supported by our current finding that latex beads coated with just the N-terminus of Als3 were able to induce endocytosis. However, it was notable that coating the latex beads with rAls3-N resulted in a much greater increase in endocytosis than adherence. Also, coating beads with rAls1-N resulted in little to no increase in adherence, even though previous studies have clearly demonstrated that Als1 is an adhesin [36,37]. The likely explanation for the relative lack of adherence of the beads is that they were coated with fragments of Als1 or Als3 that lacked the tandem repeats present in the central portion of the full-length proteins. Previously, we have found that the adhesive function of Als1 is dramatically reduced when some or all of the tandem repeats are eliminated [26]. Similarly, Oh et al. [35] found that versions of Als3 with fewer tandem repeats mediated less adherence than did versions of Als3 with more tandem repeats. In addition, Rauceo et al. [38] reported that the absence of tandem repeats reduced the binding affinity of Als5. Collectively, these results support a model in which the tandem repeats influence the conformation and/or the substrate accessibility of the N-terminal region of Als3 and thereby enhance its binding affinity for host substrates. However, even the weak binding of the N-terminal portion of Als3 to N-cadherin or E-cadherin is sufficient to induce endocytosis.

The results with the *als3*Δ/*als3*Δ null mutant and the latex beads coated with rAls3-N demonstrate that endothelial cell N-cadherin and epithelial cell E-cadherin are two host cell ligands for Als3. Furthermore, binding of the N-terminus of Als3 to either of these cadherins is sufficient to induce endocytosis. Previously, we have reported that N-cadherin is one of the endothelial cell surface proteins that mediate endocytosis of C. albicans [9]. E-cadherin is known to mediate the endocytosis of Listeria monocytogenes by epithelial cells [41], but it was not previously known to mediate the endocytosis of any fungi. Our results with C. albicans raise the interesting possibility that perhaps L. monocytogenes can utilize N-cadherin to invade endothelial cells.

Although the N-termini of Als1 and Als3 share considerable homology at the amino acid level, Als3 induced endocytosis much more efficiently than did Als1. The results of computer-assisted modeling provide a possible structural explanation for these functional differences. A striking finding was that the 3-D structures of the N-terminal regions of Als1 and Als3 are predicted to have recurrent β barrel domains and a global negative surface potential similar to the N-terminal regions of N-cadherin and E-cadherin. Even though N-terminal regions of Als1 and Als3 share general features, they are predicted to differ both in their 3-D structure and their interactions with N- and E-cadherin. While Als1 appears to favor an extended conformation, Als3 tends toward a more folded structure that forms a more distinct molecular cleft.

Our molecular modeling results also suggest that Als1 binds more weakly to N- and E-cadherin than does Als3. These results are consistent with our finding that beads coated with rAls3-N were endocytosed more avidly by CHO cells expressing N- or E-cadherin than were beads coated with rAls1-N. Computer-assisted modeling also predicted that Als1 and Als3 interact with N- and E-cadherin via different domains, and the geometry of Als3 binding to E-cadherin is similar to that of E-cadherin binding to itself. Collectively, these findings support the hypothesis that Als3 functions as a molecular mimic of mammalian cadherins, thereby facilitating C. albicans invasion of endothelial and oral epithelial cells.

The N-terminal regions of Als1 and Als3 are also predicted to share structural homology to bacterial adhesins and invasins. The conformation of Als1 is similar to that of the pfam02368 family of bacterial immunoglobulin-like domain surface protein/adhesins, which includes the invasin of Y. pseudotuberculosis [42,43]. In contrast, the cleft motif of Als3 is more similar to that of the COG4886 family of leucine-rich repeat invasins/intimins [42]. Interestingly, this family of proteins includes internalin A of L. monocytogenes, which mediates epithelial cell invasion by binding to E-cadherin [41,44].

The models presented here do not purport to specify exact interactions between cadherins and Als proteins. Defining such interactions at the atomic level requires physicochemical methods such as ultracentrifugation and x-ray crystallography. However, our findings are strengthened by the fact that the results of our modeling of cadherin–cadherin interactions were very similar to what has been found by x-ray crystallography [45].

Previously, we have reported that S. cerevisiae expressing Als1 or Als3 binds to a variety of host constituents, including endothelial cells, epithelial cells, laminin, and fibronectin [23]. In the affinity purification experiments, hyphae of the *als3*Δ/*als3*Δ null mutant failed to bind to multiple host proteins, including N-cadherin and E-cadherin. Thus, these results are consistent with the model that Als3 binds to a broad range of host substrates. However, our data also indicate that there is some specificity in the binding of Als proteins to host constituents. For example, beads coated with rAls3-N were endocytosed much more efficiently than were beads coated with rAls1-N. These results, combined with those of the molecular modeling studies, suggest that Als3 binds with greater affinity to N- and E-cadherin than does Als1. Nevertheless, it is highly likely that additional host cell ligands, other than N- or E-cadherin, also contribute to the endocytosis of *C. albicans,* and we are currently working to identify them.

For many microbial pathogens, invasion of host cells is critical for the initiation and maintenance of infection, and many of these organisms have more than one mechanism for inducing their own uptake by host cells [46]. Thus, it is logical to speculate that C. albicans has also evolved at least one Als3-independent mechanism to invade host cells. The Als family of proteins contains six members in addition to Als1 and Als3 [20–25]. Most of these proteins are known to have adhesive function. When expressed in *S. cerevisiae,* Als5 induces weak endothelial cell endocytosis similar to that induced by Als1 [23]. Als6, Als7, and Als9 do not appear to mediate significant endothelial cell endocytosis, and Als2 and Als4 have not yet been tested in this assay. While it is possible that Als3 is the only member of its family that has significant invasive function, it is also conceivable that one or more of the other Als proteins mediates invasion of other types of host cells. Thus, a detailed analysis of these proteins will likely provide further insight into the pathogenesis of hematogenously disseminated and mucosal candidiasis, and perhaps additional therapeutic targets.

## Materials and Methods

### Fungal growth conditions and strains.

For use in the experiments, all C. albicans strains were grown as blastospores in liquid YPD (1% yeast extract [Difco, http://www.bdbiosciences.com], 2% bacto-peptone [Difco], and 2% D-glucose) medium in a shaking incubator at 30 °C overnight. The blastospores were harvested by centrifugation, washed twice in phosphate-buffered saline without calcium or magnesium (PBS), and enumerated using a hemacytometer. Auxotrophic strains were grown on YPD agar supplemented with histidine, arginine, or uridine as needed. To produce hyphal phase organisms, blastospores were incubated in RPMI 1640 medium (Irvine Scientific, http://www.irvinesci.com) in a shaking incubator at 37 °C for 90–120 min as described [9].

The strains of C. albicans used in this study are listed in Table 2. Strains 7371 and 7392 were isolated from patients with AIDS who had oropharyngeal candidiasis, and were a generous gift from Thomas Patterson (University of Texas Health Sciences Center, San Antonio, Texas, United States).

**Table 2:**
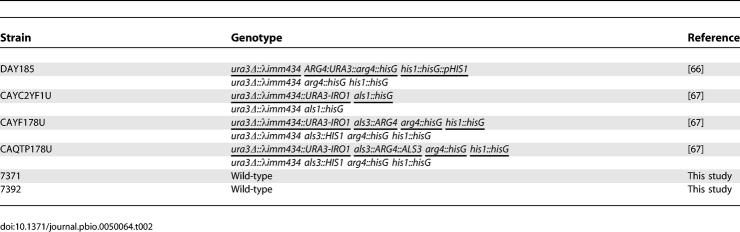
Strains of C. albicans Used in This Study

### Production and purification of rAls1-N and rAls3-N.

rAls1-N and rAls3-N were produced in S. cerevisiae as previously described [36,40]. The secreted proteins were concentrated by passing the conditioned culture supernatant through a 10-kDa filter, after which the Als proteins were affinity purified by passing the concentrate over a Ni-NTA column (Qiagen, http://www.qiagen.com). The purity of the recombinant proteins was verified by SDS-PAGE and was at least 90%.

Latex beads were coated with rAls1-N, rAls3-N, or biotinylated BSA by a minor modification of the method of Dersch and Isberg [47]. Briefly, 5 × 10^7^ fluorescent yellow-green amine–modified polystyrene latex beads (2.0 μm diameter; Sigma-Aldrich, http://www.sigmaaldrich.com) were washed first with PBS and then with coupling buffer (0.2 M Na_2_HCO_3_ [pH 8.5] and 0.5 M NaCl). The beads were then incubated with Als1, Als3 (0.5 mg/ml), or coupling buffer at 37 °C for 30 min. Next, 1% rabbit serum was added to the beads that had been coated with Als proteins, and biotinylated BSA (1 mg/ml) was added to the control beads. After the beads were incubated at 37 °C for an additional 30 min, they were sonicated briefly and then blocked by adding unlabeled BSA (to 10 mg/ml) in coupling buffer and incubating them for 1 h. Finally, the beads were then washed twice with PBS containing 10 mg BSA/ml, and suspended in PBS containing 2 mg BSA/ml. Binding of rAls1-N to the beads was verified by indirect immunofluorescence with a monoclonal antibody directed against rAls1-N [36]. Binding of rAls3-N to the beads was confirmed similarly using an affinity-purified rabbit polyclonal antiserum that been raised against rAls3-N (PickCell Laboratories, http://www.pickcell-b2b.com) and adsorbed on hyphae of the *als3*Δ/*als3*Δ mutant. Binding of biotinylated BSA to the beads was verified using Alexa 568–conjugated strepavidin (Invitrogen, http://www.invitrogen.com).

### Growth of endothelial cells, oral epithelial cells, and CHO cells.

Endothelial cells were harvested from human umbilical cord veins by the method of Jaffe et al. [48]. They were grown in M-199 medium (Invitrogen) containing 10% fetal bovine serum (FBS; Gemini Bio-Products, Inc., http://www.gembio.com), 10% defined bovine calf serum (Gemini Bio-Products, Inc.), and 2 mM L-glutamine with penicillin and streptomycin (Irvine Scientific) as described previously [4]. They were used at the second or third passage. The FaDu oral epithelial cell line, originally derived from a human pharyngeal carcinoma, was obtained from the American Type Culture Collection (http://www.atcc.org). The cells were maintained in MEM Earl's salts (Irvine Scientific) containing 10% FBS, 1 mM pyruvic acid, 2 mM L-glutamine, 0.1 mM nonessential amino acids, 100 IU/ml penicillin, and 100 IU/ml streptomycin. The OKF6/TERT-2 cell line, which had been constructed by transforming oral mucosal keratinocytes from a healthy adult with a constitutively active telomerase catalytic subunit, was kindly provided by J. Rheinwald (Harvard University, Cambridge, Massachusetts, United States) [30]. These cells were maintained in subconfluent cultures as described elsewhere [30]. To grow confluent cells, the medium was changed to a mixture containing 50% keratinocyte serum-free medium, 25% Ham's F-12, and 25% calcium- and glutamine-free DMEM (all from Invitrogen). This mixture was supplemented with 1.5 mM L-glutamine, 25 μg bovine pituitary extract/ml, and 0.2 ng epidermal growth factor/ml. CHO K-1 cells were also obtained from the American Type Culture Collection. They were grown in Ham's F-12K medium (American Type Culture Collection) supplemented with 10% FBS, penicillin, and streptomycin. All cell types were grown at 37 °C in 5% CO_2_.

### Transfection and sorting of CHO cells.

CHO cells expressing human N-cadherin and control CHO cells transfected with the empty vector pcDNA3.1 (which expressed no human cadherins) were produced as described previously [9]. CHO cells expressing human E-cadherin were generated following a similar procedure. Briefly, CHO cells were transfected with plasmid hEcad/pcDNA3, a generous gift from C. Gottardi (Northwestern University, Evanston, Illinois, United States) [49], using Lipofectamine Plus (Life Technologies, http://www.invitrogen.com) following the manufacturer's protocol. The transfectants were selected with G418 (AG Scientific, Inc., http://www.agscientific.com). Both the N-cadherin and E-cadherin transfectants were enriched by fluorescence-activated cell sorting for cells with high-level expression of these proteins [9,50]. N-cadherin– and E-cadherin–expressing cells were detected using mouse monoclonal antibodies that recognized the extracellular domains of N-cadherin (clone GC34; Sigma-Aldrich) and E-cadherin (clone HECD-1; Abcam Inc., http://www.abcam.com). The cells were used in the experiments when at least 75% of the transfectants had high level expression of either N- or E-cadherin.

### Quantification of host cell endocytosis and binding of C. albicans or latex beads.

The number of organisms or latex beads that were endocytosed or cell-associated with the various host cells was determined using our standard differential fluorescence assay [5,7,9,51]. The host cells were grown to 95% confluency onto 12-mm diameter coverslips coated with fibronectin in 24-well tissue-culture plates. They were incubated with 10^5^ C. albicans hyphae in RPMI 1640 medium. After 45 min, the cells were rinsed twice with Hank's balanced salt solution (HBSS; Irvine Scientific) in a standardized manner and then fixed with 3% paraformaldehyde. In experiments performed with *C. albicans,* the adherent but nonendocytosed organisms were labeled with rabbit polyclonal anti–C. albicans antibodies (Biodesign International, http://www.biodesign.com) that had been conjugated with Alexa 568 (Invitrogen), which fluoresces red. Next, the cells were permeablized with 0.5% Triton X-100 (Sigma-Aldrich) in PBS, and then the cell-associated organisms (the endocytosed plus nonendocytosed organisms) were labeled with the anti–C. albicans antibodies conjugated with Alexa 488 (Invitrogen), which fluoresces green. The coverslips were viewed using an epifluorescent microscope, and the number of endocytosed organisms was determined by subtracting the number of nonendocytosed organisms (which fluoresced red) from the number of cell-associated organisms (which fluoresced green). At least 100 organisms were examined on each coverslip, and the results were expressed as the number of endocytosed or cell-associated organisms per high-powered field.

Experiments investigating the endocytosis and adherence of the yellow-green fluorescing latex beads were performed similarly, except that 3 × 10^5^ beads were added to each well. The adherent, nonendocytosed control beads coated with biotinylated BSA were labeled with strepavidin Alexa 568. The adherent beads coated with either rAls1-N or rAls3-N were detected by indirect immunofluorescence using rabbit polyclonal anti-Als1 antibodies followed by Alexa 568–conjugated goat anti-rabbit antibodies.

### Affinity purification of host cell surface proteins that bind to C. albicans.

Membrane proteins from host cells were labeled with biotin and then isolated as described previously [9]. Briefly, endothelial or oral epithelial cells were incubated with EZ link Sulfo NHS- LC biotin (0.5 mg/ml; Pierce, http://www.piercenet.com) in PBS with calcium and magnesium (PBS^++^) for 12 min at 37 °C in 5% CO_2_. After extensive rinsing with cold PBS^++^, the cells were scraped from the tissue-culture dishes and collected by centrifugation at 500*g.* Next, the cells were lysed by incubating them for 20 min on ice in PBS^++^ containing 5.8% octyl-glucopyranoside (wt/vol; Sigma-Aldrich) and protease inhibitors (1 mM PMSF, 1μg/ml pepstatin A, 1μg/ml leupeptin, and 1μg/ml aprotinin). The lysate was clarified by centrifugation at 5000*g* for 5 min at 4 °C, after which the supernatant was centrifuged at 100,000*g* for 1 h at 4 °C. The resultant supernatant was stored in aliquots at −80 °C. The protein concentration was determined by the Bradford assay (Bio-Rad Laboratories, http://www.bio-rad.com).

The host cell membrane proteins that bound to C. albicans hyphae were isolated by a minor modification of our previously described affinity purification procedure [9]. In brief, 250 μg of the biotinylated membrane proteins in PBS^++^ containing 1.5% octyl-glucopyranoside and protease inhibitors was incubated with 2 × 10^8^ C. albicans hyphae on ice for 1 h. The unbound proteins were removed by extensive rinsing in the same buffer. Next, the proteins that had bound to the hyphae were eluted with 6 M urea. The eluted proteins were separated by SDS-PAGE on an 8% gel and detected by immunoblotting with an anti-biotin murine monoclonal antibody (clone BN-32; Sigma-Aldrich), an anti-N-cadherin murine monoclonal antibody (clone 32; Transduction Laboratories, http://www.bdbiosciences.com), or the anti-E-cadherin murine monoclonal antibody. The membranes were then incubated with an appropriate horseradish peroxidase–conjugated secondary antibody, and the bands were visualized using enhanced chemiluminescence (Pierce).

### Immunofluorescence detection of cadherin localization.

Indirect immunofluorescence was used to determine whether endothelial cell N-cadherin and oral epithelial cell E-cadherin colocalized with hyphae of the various strain of C. albicans as previously described [9]. Endothelial or FaDu oral epithelial cells were grown onto 12-mm diameter glass coverslips as in the endocytosis experiments. The cells were infected with 2.5 × 10^5^ blastospores of C. albicans for 90 min. During this time, the organism germinated and formed hyphae. Next, the cells were fixed and permeabilized with 3% paraformaldehyde containing 0.5% triton X-100 in PBS^++^. They were rinsed once with 1% BSA in PBS and blocked with 5% goat serum in PBS. To visualize F-actin, the cells were incubated with Alexa 568 phalloidin. After extensive rinsing, the cells were incubated with either the anti-E-cadherin murine monoclonal antibody or an anti-N-cadherin monoclonal antibody (clone 32), rinsed and then incubated with polyclonal goat anti-mouse antibodies conjugated with Alexa 488. The C. albicans cells were then labeled by incubation with the rabbit anti–C. albicans antiserum conjugated with Alexa 633 (Invitrogen). The coverslips were mounted inverted, and imaged by confocal scanning laser microscopy. Images of the organisms and host cells were acquired, and three to five optical sections were stacked along the *z*-axis to produce the final images.

### Determining the effects of saliva on C. albicans interactions with oral epithelial cells.

To determine the effects of saliva on the interactions of C. albicans with FaDu oral epithelial cells, the organisms were germinated in RPMI 1640 medium to form hyphae as described above. Next, they were killed by incubation in 0.02% thimerosal (2-[ethylmercuriomercapto] benzoic acid sodium salt) in HBSS at 37 °C for 1 h [5] and then rinsed extensively. This method of killing was used to minimize changes in the cell wall [52]. Uninduced saliva was collected from normal volunteers. It was centrifuged at 500*g* and then sterilized by passage through a 0.22-μm filter. The killed organisms were incubated in 20% saliva in RPMI 1640 medium at 37 °C for 1 h, after which they were rinsed twice with HBSS. Control organisms were incubated in RPMI 1640 medium without saliva in parallel. The ability of the saliva-coated organisms to bind to oral epithelial cell proteins and induce endocytosis was determined as described above.

### Detection of Als3 expression on the C. albicans surface.

Flow cytometry was used to analyze the surface expression Als3 on hyphae of the various strains using a minor modification of our previously described method [23]. Briefly, hyphae of the different strains of C. albicans were fixed in 3% paraformaldehyde and blocked with 1% goat serum. The hyphae were then incubated with either a rabbit polyclonal antiserum raised against rAls3-N or purified rabbit IgG. After extensive rinsing, the cells were incubated with a goat anti-rabbit secondary antibody conjugated with Alexa 488. The fluorescent intensity of the hyphae was measured using a FACSCaliber flow cytometer (Becton Dickinson, http://www.bd.com). Fluorescence data for 10,000 cells of each strain were collected.

### Structural and functional modeling of rAls1-N and rAls3-N.

The structures of rAls1 and Als3 were modeled using a combination of knowledge- and energy-based methods to avoid limits of any single method in assessing folding of multiple Als domains. Homolog searches were conducted on 100–200 residue blocks of each Als sequence. The domains identified were joined, and the most likely domain configuration was determined by Monte Carlo searches of φ and ψ angles for connecting regions. The domains presented were the consensus of folds identified by Composer [53], MatchMaker [54], GeneFold [55], Robetta [56], Fugue [57], and Swiss-Model [58]. The AMBER99 force fields were used throughout these studies to optimize models. AMBER8 [59] and SYBYL 7.0–7.2 (Tripos Inc., http://www.tripos.com) were implemented on multiple graphics workstations (Sun Microsystems, Inc., http://www.sun.com; and SGI, Inc., http://www.sgi.com) using LINUX and IRIX operating systems. In addition to the semi-automated procedures above, backbone trajectories were obtained using FASTA searches of the Research Collaboratory for Structural Bioinformatics (RCSB) protein databank (http://www.rcsb.org). Side chains of the target rAls1 and rAls3 models were refined by molecular dynamics and minimization of strain energies, while retaining backbone trajectory by fixing positions of the peptide backbone atoms. The torsion angles of all peptide bonds were adjusted to 180° ± 15°, with minimal constraints. Where appropriate, selected model constraints applied a 0.4-kJ penalty to the Ramachandran φ and ψ angles to enforce regions of predicted α and β structures. Final global energy minimizations were performed for each model after removal of all constraints. Finally, the physicochemical properties of the peptides were visualized by MOLCAD [60] as implemented in SYBYL 7.0–7.2 and HINT [61]. In these utilities, the physical properties of the peptides are superimposed upon the backbone trajectory, or projected onto the water-accessible surface of the molecules.

### Computational modeling of Als protein–cadherin interactions.

Models of the N-terminal regions of Als1 and Als3 generated as above were then examined for predicted interactions with E-cadherin or N-cadherin [62] in silico using the program Hex 4.5 [63,64]. The structures of these cadherins have been determined to a resolution of 2 Å by x-ray crystallography [45]. To enhance the predictive accuracy of modeling and evaluate all probable interactions, multiple docking simulations were performed from different starting positions. For example, in a systematic approach, docking was initiated with the target cadherin located next to each of the major Als N-terminal domains. The search range was set to 60 Å using full rotation mode and a 0.75 Å step size with two substeps. Initial searches were based on shape and electrostatics; post-processing was based on 3-D shape and surface topography. Post-processing with molecular mechanics or molecular mechanics minimization (0.75 Å step size, two substeps) gave equivalent results. As a comparator to Als-cadherin interactions, the self-association of two N-terminal domains of E-cadherin and N-cadherin was also assessed. To assess the validity of the hex algorithms applied, we separated the two E-cadherin molecules, the structures of which are contained in the Protein Data Bank (http://www.pdb.org) [45]. The two peptides were then redocked using the approach described above, initiated from multiple orientations of the peptides. In every case, the best dock was clearly discernable by the energy score. Visually, E-cadherin redocking was virtually indistinguishable from that of the reported structure determined by crystallography [45]. Quantitatively, redocked E-cadherins had a root mean square deviation of < 1 Å deviation from the crystal structure. No attempt was made to optimize the output because the crystal packing forces may account in part for any differences between published structures [45] and hex algorithm model output. Nonetheless, docking parameters (Table 1 **)** varied less than 10% between the experimental and redocked E-cadherins (unpublished data).

### Statistical analyses.

The data were evaluated using analysis of variance, and *p-*values ≤ 0.05 were considered significant.

## Supporting Information

Figure S1 C. albicans Interacts with OKF6/TERT-2 Oral Epithelial Cells Similarly to FaDu Oral Epithelial Cells (A) Number of hyphae of the indicated C. albicans strains that were endocytosed by and cell-associated with the OKF6/TERT-2 oral epithelial cell line after a 45-min incubation. The data are expressed as a percentage of the results with wild-type organisms, and are the mean ± SD of three experiments, each performed in triplicate. *, *p* < 0.002 compared to the wild-type strain and the *als3*Δ/*als3*Δ::*ALS3*-complemented strain.
(B and C) Immunoblot of biotinylated FaDu and OKF6/TERT-2 cell surface proteins eluted from the wild-type and *als3*Δ/*als3*Δ null mutant strains of C. albicans. The same blot was probed with antibodies against biotin (B) or E-cadherin (C). Arrow indicates the band corresponding to E-cadherin.
(186 KB PDF)

Figure S2 Saliva Has No Effect on the Interactions of C. albicans with Oral Epithelial Cells (A) Live, wild-type C. albicans hyphae and thimerosal-killed hyphae that had been incubated in either the presence or absence of 20% saliva were added to FaDu oral epithelial cells. After a 45-min incubation, the number of organisms that were endocytosed by and cell-associated with the epithelial cells was determined. The data are expressed as a percentage of the results with live organisms without saliva, and are the mean ± SD of three independent experiments, each performed in triplicate.
(B and C) Immunoblot of biotinylated FaDu cell surface proteins eluted from either live hyphae or killed hyphae that had been incubated in the presence or absence of 20% saliva. The same blot was probed with antibodies against biotin (B) or E-cadherin (C).
(805 KB PDF)

Figure S3 Specificity of the Anti-N-Cadherin and Anti-E-Cadherin Monoclonal Antibodies Confocal micrographs of C. albicans hyphae that were incubated with the anti-N-cadherin (A) or anti-E-cadherin (C) monoclonal antibodies in the absence of host cells. (B) The same microscopic field as in (A) showing C. albicans hyphae, which were stained with the anti–C. albicans antiserum. (D) The same microscopic field as in (C), showing the C. albicans hyphae.
(904 KB PDF)

### Accession Numbers

The Protein Data Bank (http://www.pdb.org) accession numbers for the structures mentioned in this article are E-cadherin (1EDH) and N-cadherin (1NCJ); the Candida Genome Database (http://www.candidagenome.org) accession numbers for the genes mentioned in this article are *ALS1* (orf19.5741) and *ALS3* (orf19.1816).

## Abbreviations

BSA: bovine serum albumin
CHO: Chinese hamster ovary
PBS: phosphate-buffered saline without calcium or magnesium
PBS^++^: PBS with calcium and magnesium
rAls1-N: recombinant N-terminal portion of Als1
